# Mechanisms of resistance to trastuzumab deruxtecan in breast cancer elucidated by multi-omic molecular profiling

**DOI:** 10.1038/s41523-025-00868-y

**Published:** 2025-12-20

**Authors:** George W. Sledge, Joanne Xiu, Reshma L. Mahtani, Ana C. Sandoval Leon, Funda Meric-Bernstam, Jennifer R. Ribeiro, Ninad N. Kulkarni, Dileep R. Rampa, Jangsoon Lee, Naoto T. Ueno, Matthew J. Oberley, Milan Radovich, David B. Spetzler

**Affiliations:** 1 Caris Life Sciences, Phoenix, AZ USA; 2 Miami Cancer Institute, Miami, FL USA; 3 MD Anderson Cancer Institute, Houston, TX USA; 4 University of Hawai’i Cancer Center, Honolulu, HI USA

**Keywords:** Cancer, Drug discovery, Oncology

## Abstract

Trastuzumab deruxtecan (T-DXd) is an antibody-drug conjugate successfully used to treat HER2-low and HER2-positive metastatic breast cancer, but resistance consistently develops. Using multivariate Cox proportional hazards in a real-world cohort of 2,799 patients with breast cancer, we aimed to identify clinically relevant T-DXd resistance mechanisms. In patients with samples collected prior to T-DXd treatment, higher expression of *ERBB2* (HER2) and lower expression of *ABCC1* (an ATP-binding cassette transporter involved in drug efflux) were significantly associated with longer T-DXd-related overall survival (OS); *ABCC1* predicted OS independently of HER2. Furthermore, mutations in several genes were enriched in post-T-DXd samples compared to unmatched T-DXd-naïve samples, including *ERBB2*, *NFE2L2* (a transcriptional activator of *ABCC1*), and *KEAP1* (a negative regulator of NFE2L2), indicating plausible resistance mechanisms related to HER2 target levels and ABCC1-mediated drug efflux. Identifying such resistance mechanisms might lead to improved methods of precision oncology and novel therapeutic approaches to overcome resistance.

## Introduction

**Fig. 1:**
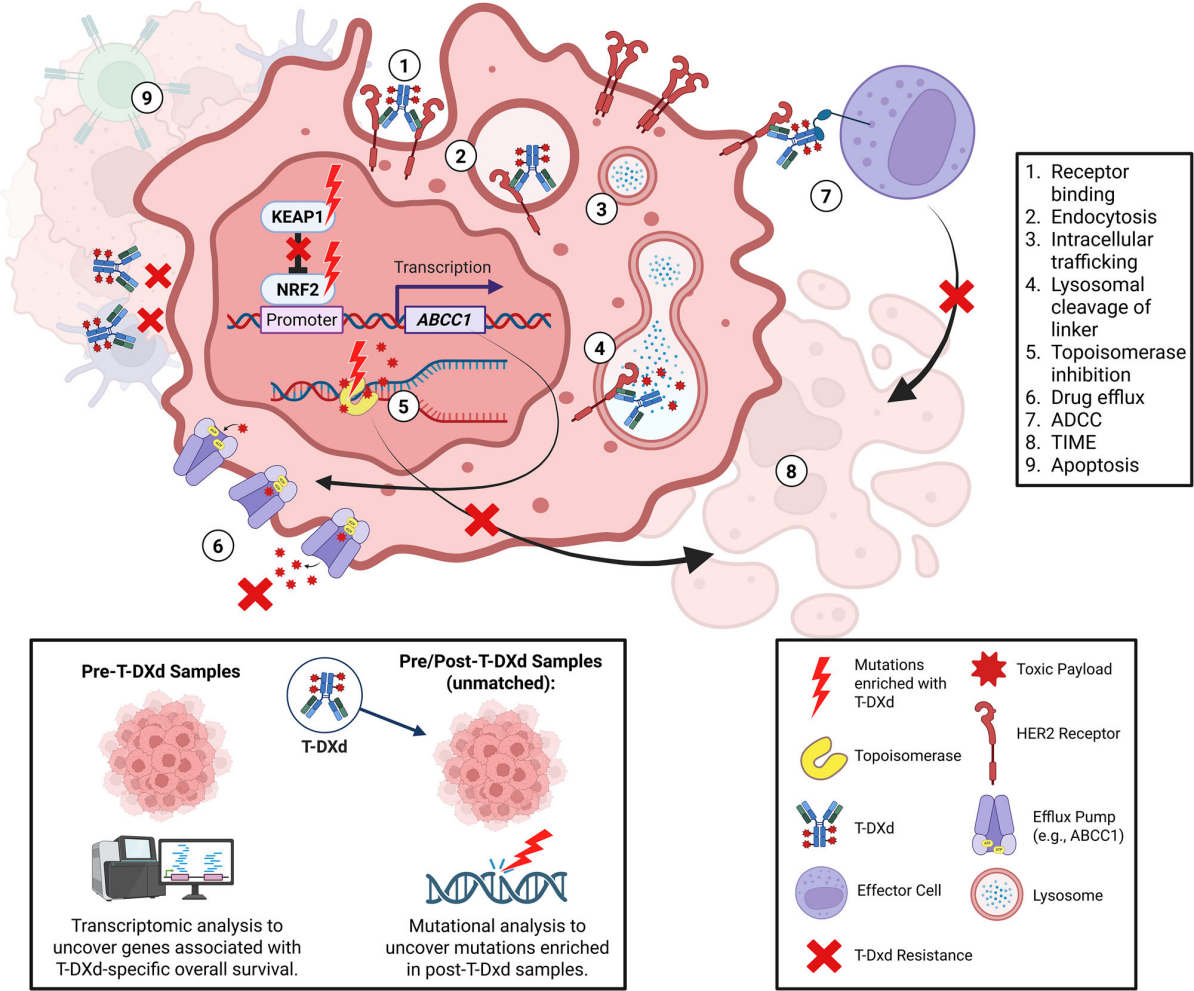
Graphical study schema and proposed resistance mechanisms. Created in BioRender. Ribeiro, J. (2026) https://BioRender.com/vr77in9.

Trastuzumab deruxtecan (T-DXd) is an antibody-drug conjugate (ADC) widely used as a treatment for HER2-low and HER2-positive metastatic breast cancer. Preclinical studies have identified multiple potential mechanisms of resistance that can develop at various points in the course of ADC trafficking through cancer cells—from receptor binding, to internalization and processing of the ADC, to efflux of the payload out of the cell, as well as antibody-dependent cellular cytotoxicity (ADCC)^1–3^ (Fig. 1). Despite the knowledge of T-DXd resistance mechanisms generated through preclinical studies, there are few large population-based studies that have explored resistance to this therapy^4^.

The availability of real-world data from insurance claims, combined with whole transcriptome sequencing, offers a unique opportunity to evaluate resistance mechanisms across key pathways in an unbiased and comprehensive manner. In this study, we integrated next-generation sequencing (NGS), immunohistochemistry (IHC), and claims data to identify clinically relevant mechanisms of resistance to T-DXd. Our aim was to identify genes involved in key processes of T-DXd’s mechanism of action that are independently predictive of T-DXd benefit, while also considering the impact of the tumor microenvironment^5,6^. Acknowledging the potential effect the treatment might have on gene expression profiles, we focused our discovery work on specimens collected prior to the start of T-DXd. In addition, we examined mutational events occurring after treatment with T-DXd to evaluate the role of selective evolutionary pressures produced by T-DXd.

## Results

### Patient characteristics

Among the 2799 tumors investigated, the majority (86.5%, *n* = 2420) were collected before the initiation of T-DXd treatment. The median length of time between tissue collection and treatment start for these patients was 15.5 months (range: 0.1–192 months). Approximately 14% of patients had received TDM1 prior to T-DXd. Using HER2 IHC/CISH criteria, approximately one-third of patients had HER2-low tumors, while those with HER2-positive and HER2-ultra-low tumors each comprised about 16% of the cohort. Surprisingly, 18% of patients treated with T-DXd had HER2-null tumors, which might be related to tumor heterogeneity, discrepancies in HER2 testing between community centers and centralized laboratories, or off-label use of T-DXd. Out of the total cohort, 13.5% (*n* = 379) of samples were collected after the initiation of T-DXd. The median length of time between treatment start and tissue collection for these patients was 10.5 months (range: 0.1–44.5 months). There were no matched samples in the analysis (Table 1).

**Table 1:** Clinico-demographic characteristics of T-DXd-treated breast cancer cohort (*N* = 2799)

	Category	Pre-T-DXd	Post-T-DXd	Total
Age	Median	58	59	59
	IQR	49–67	51–67	50–67
Sex, *N*	Female	2394	375	2769
	Male	26	4	30
HER2 status, *N* (%)	HER2-null	436 (18%)	68 (17.9%)	504 (18%)
	HER2-ultra-low	415 (17.1%)	33 (8.7%)	448 (16%)
	HER2-low	812 (33.6%)	142 (37.5%)	954 (34.1%)
	HER2-positive	396 (16.4%)	50 (13.2%)	446 (15.9%)
	Other/unknown	361 (14.9%)	86 (22.7%)	447 (16%)
HR status, *N* (%)	HR-negative	595 (24.6%)	95 (25.1%)	690 (24.7%)
	HR-positive	1,502 (62.1%)	202 (53.3%)	1,704 (60.9%)
	Unknown	323 (13.3%)	82 (21.6%)	405 (14.5%)
Specimen site, *N* (%)	Breast	661 (27.3%)	37 (9.8%)	698 (24.9%)
	Liver	517 (21.4%)	153 (40.4%)	670 (23.9%)
	Lymph node	284 (11.7%)	26 (6.9%)	310 (11.1%)
	Bone	254 (10.5%)	19 (5%)	273 (9.8%)
	Lung	160 (6.6%)	28 (7.4%)	188 (6.7%)
	Other	544 (22.5%)	116 (30.6%)	660 (23.6%)
Test platform, *N* (%)	RNA (WTS)	1714 (71%)	115 (30%)	1829 (65%)
	DNA (NGS)	2069 (85%)	362 (96%)	2432 (87%)
Total, *N*		2420	379	2799

### Association of key genes with overall survival following T-DXd

The mechanism of action for T-DXd has been extensively investigated in preclinical and clinical studies, leading to a substantial increase in knowledge about individual pathways in recent years^7^. We compiled a set of 96 transcriptomic features (RNA signatures and gene expressions) across 11 curated pathways/functions: tumor immune microenvironment (TIME), ATP-binding cassette (ABC) transporter, ADCC pathway, cytoskeleton organization, endocytosis, intracellular trafficking, lysosome pathway, prognostic markers, target dimerization partners, topoisomerases, and tubulins (Supplementary Table S2). We performed multivariate Cox proportional regression analysis to assess the association of these features with T-DXd-specific overall survival (OS) in 1714 pre-T-DXd tumors with RNA expression data available.

**Fig. 2:**
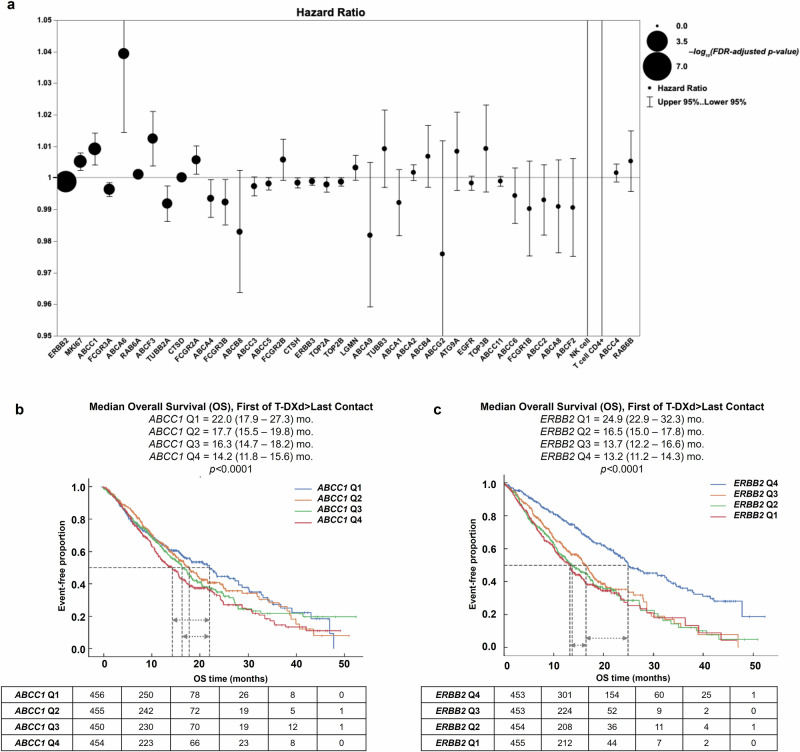
Multivariate analysis using Cox-Proportional Regression Model in pre-treatment specimens identifies *ABCC1* as highly associated with T-DXd associated overall survival. Expression level was treated as a continuous variable in the discovery work. **a** Hazard ratios are indicated by the circles, with upper and lower 95% confidence intervals represented by the whiskers. Statistical significance (log_10_ FDR-adjusted p-value) is indicated by the size of the circles, with larger circles indicating greater significance. Kaplan–Meier evaluation of *ABCC1* (**b**), and *ERBB2* (**c**) expression quartiles (Q4: highest expression, Q1: lowest expression) and association with T-DXd-specific overall survival. OS was calculated from start of treatment with T-DXd to last patient contact using insurance claims data. *p* value was calculated using log-rank test.

Six genes were identified with significant associations between their expression levels and T-DXd OS (*q* < 0.05, Fig. 2a). Specifically, higher expression levels of *ERBB2* (HER2) and *FCGR3A* (Fc gamma receptor IIIa) were each associated with improved T-DXd-specific OS [hazard ratio, i.e., HR associated with each unit (TPM) change of RNA < 1]. Conversely, increased expression of *MKI67* (Ki-67), *ABCC1* (ATP Binding Cassette Subfamily C Member 1, also known as multidrug resistance-associated protein 1 or MRP1), *ABCA6* (ATP Binding Cassette Subfamily A Member 6), and *RAB6A* (Ras-related protein Rab-6A) correlated with worse T-DXd-specific OS. Stratifying the entire WTS-tested cohort based on expression quartiles of the six genes revealed an increase in T-DXd-specific OS with the highest *ERBB2* expression and a stepwise decrease in T-DXd-specific OS with increased *MKI67* and *ABCC1* expression (Fig. 2b, c). Kaplan–Meier analysis showed no difference in T-DXd-specific OS according to *FCGR3A*, *ABCA6*, and *RAB6A* expression (Supplementary Fig. S1).

Given the generic role of Ki-67 as a prognostic marker of proliferation (supported by our findings that *KI67* is prognostic regardless of T-DXd treatment (Supplementary Fig. S2), we focused on the ABC transporter *ABCC1* as a potential predictor of resistance to T-DXd. We examined the correlation of *ABCC1* with all other 95 features and found that *ABCB7, ABCE1*, and *ABCF2* expression significantly correlated with *ABCC1* [Spearman *ρ* > 0.6 (0.60–0.64)]. Adding interactions of *ABCC1* with these three genes showed that *ABCC1* remained an independent predictor of T-DXd-specific OS (Supplementary Table S3).

### Expression of ERBB2 and ABCC1 across HER2 groups

**Fig. 3:**
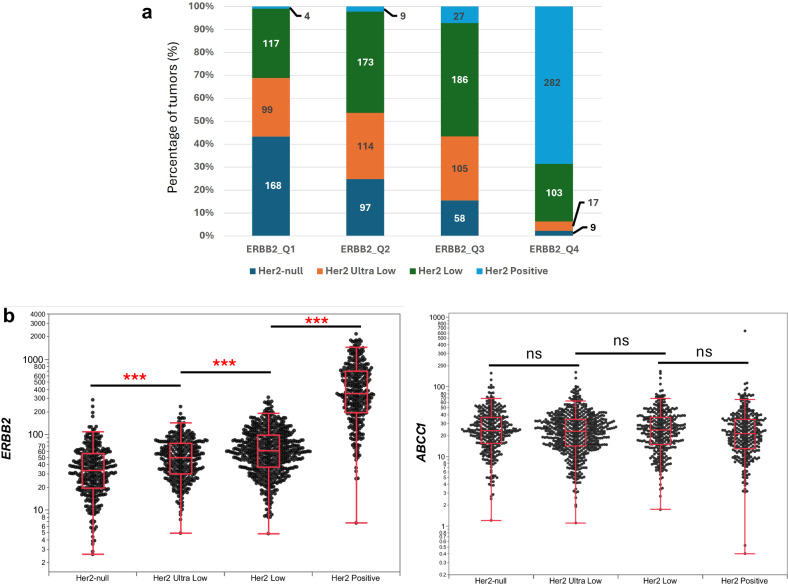
*ERBB2* and *ABCC1* RNA expression according to HER2 status. **a** Distribution of HER2 status (HER2-null, HER2-ultra-low, HER2-low and HER2-positive categories) in *ERBB2* quartiles (Q4: highest expression, Q1: lowest expression). Numbers of cases are shown within bars. **b** HER2 (*ERBB2)* expression (TPM) was significantly associated with HER2 status (left panel) while *ABCC1* expression was independent of HER2 status (right panel).

As the expression of *ERBB2* and *ABCC1* was top predictors of T-DXd-associated OS, we further investigated their expression patterns in public datasets and in our dataset. Using the cBioPortal^8,9^, we found that *ABCC1* was among the most frequently altered (amplifications and mRNA-high expression relative to diploid) and highly expressed ABC transporter at the RNA level in the Cancer Genome Atlas (TCGA) invasive breast cancer cohort, and its protein level correlated modestly with transcript expression (*r* ≈ 0.4) (Supplementary Fig. S3a–c). In our cohort, HER2-positive tumors were predominantly enriched among tumors with the top quartile expression of *ERBB2*. Conversely, HER2-low tumors showed a wide range of *ERBB2* expression, with more equal distribution across the *ERBB2* expression quartiles (>20% in each category). Similarly, HER2-ultra-low tumors had variable *ERBB2* expression, although they comprised a smaller proportion of the highest *ERBB2* expression group (Fig. 3a). Expectedly, we saw that *ERBB2* TPM was significantly associated with ASCO/CAP HER2 categories^10^ as described in Supplementary Table S1 (Fig. 3b). Conversely, *ABCC1* expression showed no association with HER2 status and remained consistent across all categories (Fig. 3b), which we also observed in TCGA cohort (Supplementary Fig. S3c).

### Association of ERBB2 and ABCC1 with overall survival following T-DXd

**Fig. 4:**
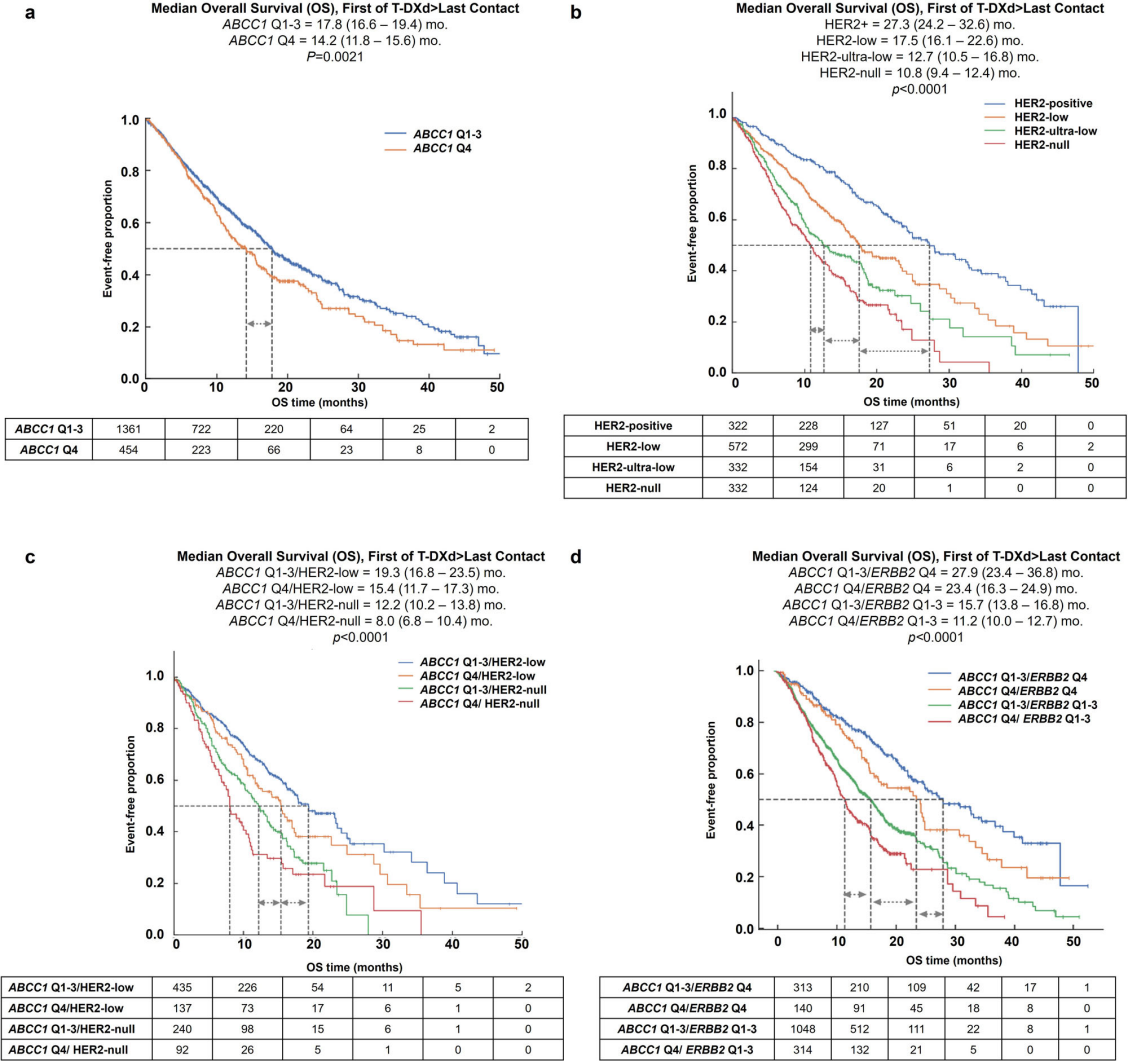
Association of *ABCC1* gene expression and HER2/*ERBB2* with T-DXd associated overall survival. Kaplan–Meier curves show median T-DXd-specific overall survival based on **a** *ABCC1* high (Q4) vs. *ABCC1* low (Q1-Q3); **b** HER2 status; **c** a combination of HER2 status and *ABCC1* expression; and **d** a combination of *ERBB2* expression and *ABCC1* expression.

Baseline clinical and demographic characteristics of the patient cohort stratified by *ABCC1* expression quartiles are shown in Supplementary Table S5. Using Kaplan–Meier survival analysis, we observed that high expression of the drug efflux pump *ABCC1* was significantly associated with decreased OS following T-DXd, implicating increased efflux of the cytotoxic deruxtecan payload may reduce T-DXd efficacy. As shown in Fig. 2c, patients in the bottom quartile of *ABCC1* expression (Q1) had significantly improved OS compared to the top two quartiles (Q3 and Q4) of expression and numerical increase from Q2 A series of thresholds of *ABCC1* expression were tested using 5 percentile increments, which justified using the top quartile as an optimal cutoff to achieve the most significance, meaningful hazard ratios, and optimal patient cohort sizes (Supplementary Table S4). Stratifying patients into *ABCC1*-High (Q4) and *ABCC1*-Low (*ABCC1* Q1-3) revealed that the *ABCC*1-Low group had a significantly better OS (Fig. 4a).

As expected, ASCO/CAP-guided HER2 categorization (using IHC and CISH) was strongly associated with T-DXd associated OS (Fig. 4b). Notably, stratifying into *ABCC1*-High and *ABCC1*-Low groups further predicted patient outcome within each HER2 category to some degree (Supplementary Fig. S4), particularly for patients with HER2-low and HER2-null tumors (Fig. 4c). Similarly, when patients were stratified using a combination of *ERBB2* RNA expression and *ABCC1* expression (Fig. 4d), a clear improvement in OS was seen for patients with low *ABCC1* expression among both *ERBB2*-Low and *ERBB2*-High groups. Together, these findings highlight the importance of *ABCC1* status in predicting treatment outcomes, independently of HER2/*ERBB2*.

To confirm that *ABCC1* is a biomarker for T-DXd and not simply prognostic, we leveraged a large cohort of breast tumors with *ABCC1* expression and outcome data. All patients were included in this cohort regardless of treatment. When analyzing OS calculated from tissue collection to last contact, there was no difference in OS between *ABCC1*-high versus *ABCC1*-low groups (HR = 1.03 (95% CI: 0.99–1.06), *p* = 0.152). When patients were stratified into quartiles based on *ABCC1* expression, OS also did not show any incremental increase with increasing expression (Supplementary Fig. S5).

### Post-trastuzumab deruxtecan molecular events

**Fig. 5:**
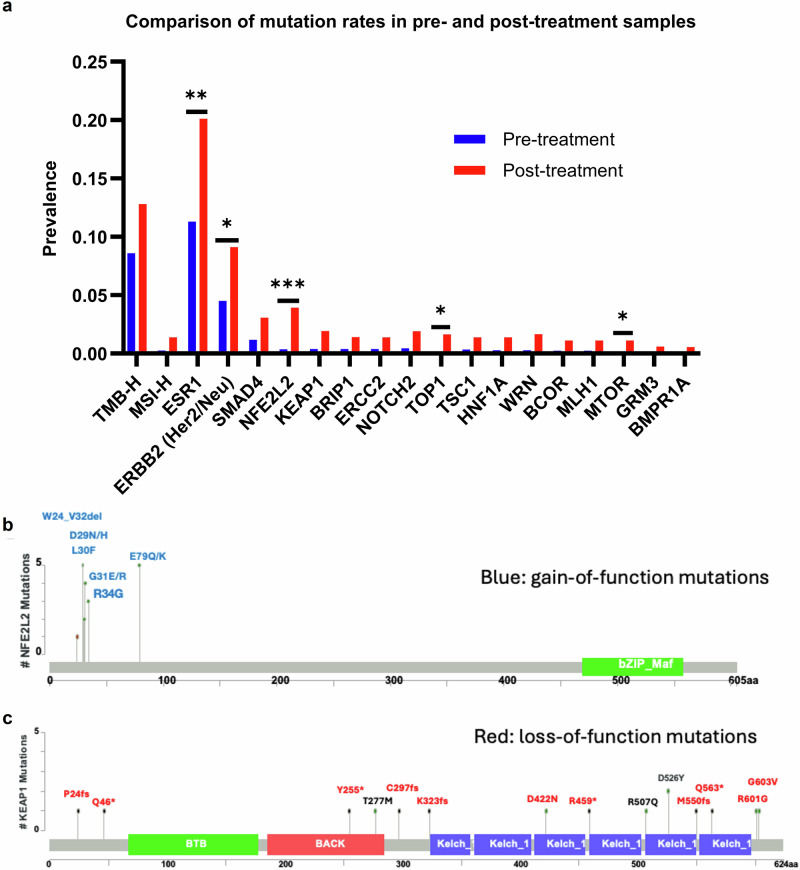
Mutational events following T-DXd treatment. **a** Comparison of pre- and post-treatment molecular profiles. All mutations shown have *p* < 0.05 (unadjusted *p* value). *: *q* < 0.05; **: *q* < 0.005; ***: *q* < 0.0005. **b** Lollipop graphs showing gain-of-function mutations of *NFE2L2* and **c** loss-of-function mutations in *KEAP1*.

To identify the development of potential T-DXd resistance mechanisms, we compared molecular characteristics of tumors treated with T-DXd prior to tissue collection for NGS testing with tumors treated with T-DXd after tissue collection. As expected, *ABCC1* expression was significantly higher in post-treatment samples compared to pre-treatment samples (median: 27 TPM vs. 22 TPM, *p* < 0.001). In contrast, we did not observe a similar post-treatment increase in a group of patients treated with trastuzumab, suggesting that the upregulation of *ABCC1* is T-DXd-specific (Supplementary Fig. S6). In addition, potentially important mutational events occurring with significantly increased frequency in post-T-DXd samples are shown in Fig. 5a. In decreasing order, the most frequent mutational events occurred in *ESR1* (*q* < 0.005), *ERBB2* (*q* < 0.05), *SMAD4* (*p* = *0.01; q* = 0.3) *NFE2L2* (*q* < 0.0005), *TOP1* (*q* < 0.05) and *KEAP1* (*q* = 0.074). The mutational landscape of all pre- and post-T-DXd tumors is shown in Supplementary Fig. S7.

Mutations in *ERBB2*, such as those that inhibit trastuzumab binding, can induce resistance to HER2-targeted therapies, including T-DXd^11–13^. In our cohort, 78% of *ERBB2* mutations occurred in the kinase domain, with similar proportions in pre- and post-treatment samples, implicating *ERBB2* alterations as one potential resistance mechanism in addition to HER2 cell surface levels (Supplementary Fig. S8a). Likewise, mutations in *TOP1* (topoisomerase 1) have also been reported to promote cross-resistance to ADCs in patients with MBC^14^. We similarly observed more *TOP1* mutations in post-T-DXd-treated samples compared to T-DxD-naïve samples in our cohort (Fig. 5a and Supplementary Fig. S8b).

*ESR1* mutations are known to be associated with prolonged aromatase inhibitor therapy in ER-positive breast cancer^15^, so the increased prevalence of *ESR1* mutations here may merely represent a population with longer exposure to endocrine therapy. Consistent with this hypothesis, *ESR1* mutations were highly concentrated in ER-positive tumors (18%, *n* = 260/1449), with far fewer being seen in ER-negative tumors (0.3%, *n* = 2/662), which are not treated with endocrine therapy. To further investigate this hypothesis, we summarized other pre-collection therapies that the patients received that may have exerted mutational pressures (Supplementary Table S5). Approximately one quarter of patients across all quartiles of *ABCC1* expression received hormonal therapy prior to tissue collection, suggesting the plausibility of *ESR1* mutations as an effect of this exposure.

*NFE2L2* (also known as NRF2) is a transcriptional regulator that upregulates *ABCC1*, while *KEAP1* is a negative regulator of NFE2L2^16,17^. Thus, the gain-of-function mutations in *NFE2L2* that we observed (Fig. 5b) are predicted to cause upregulation of *ABCC1*, while the loss-of-function mutations that we observed in *KEAP1* (Fig. 5c) are predicted to promote increased activity of NFE2L2, also causing upregulation of *ABCC1*. We did not observe a significant difference in *ABCC1* expression between tumors with and without *NFE2L2* or *KEAP1* mutations, but this analysis was not statistically powered (Supplementary Fig. S9).

### Validation in ADC-resistant breast cancer cells

**Fig. 6:**
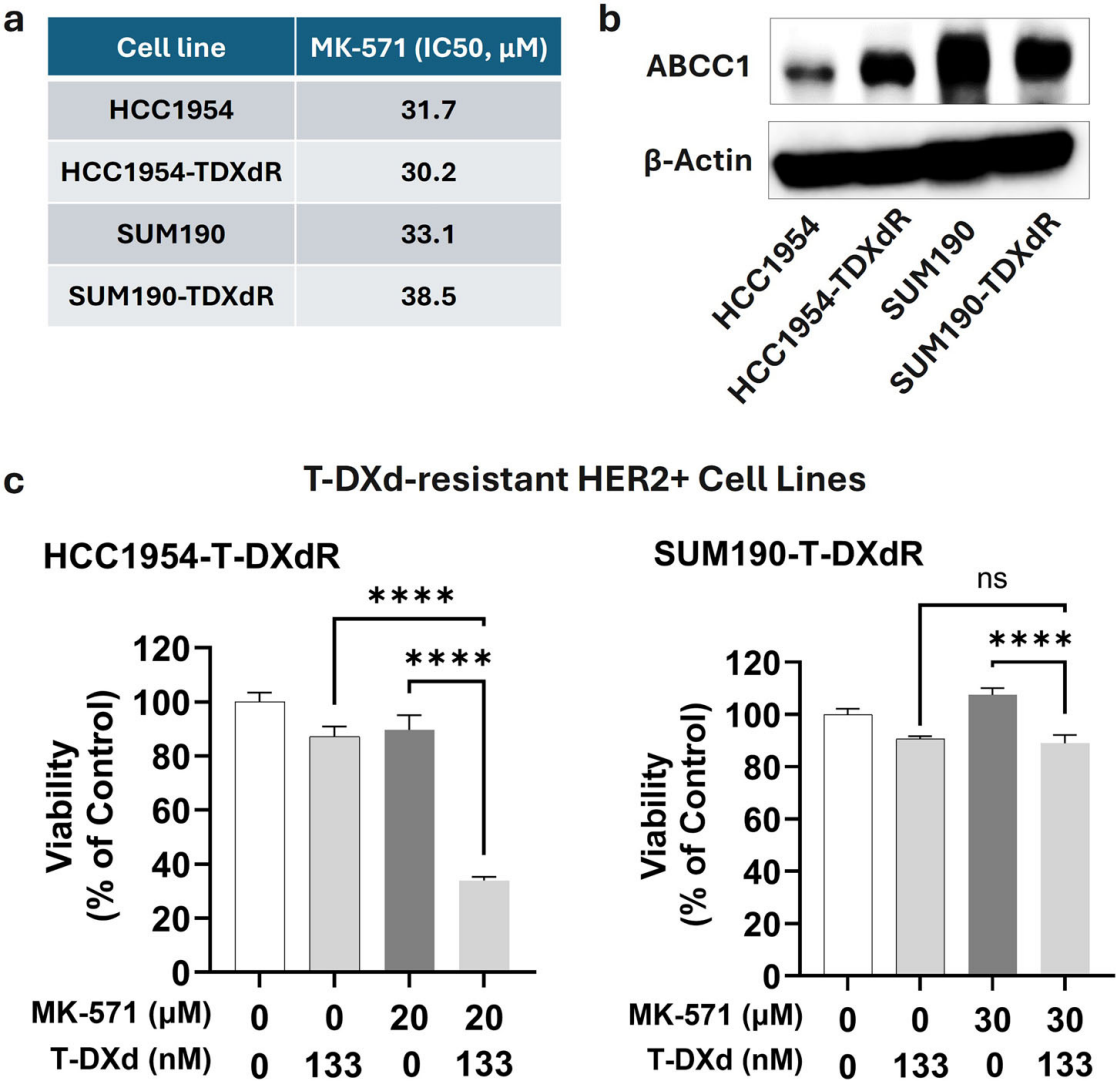
Transporter inhibitor enhances the efficacy of T-DXd in ADC-resistant breast cancer cell lines. **a** IC50 values for MK-571 in parental and resistant cell lines. **b** Western blot of ABCC1 levels in parental and resistant cell lines. β-actin was used as a loading control. Uncropped blots are shown in Supplementary Fig. S10. **c** MTS cell viability assay. Parental and resistant cell lines were treated with MK-571, T-DXd, or MK-571 + T-DXd for 10 days prior to MTS assay.

To evaluate the potential of ABCC1 inhibition in reversing ADC resistance, we first assessed the sensitivity of previously characterized HER2+ parental and T-DXd-resistant breast cancer cell lines^18^ to MK-571. IC50 values for MK-571 ranged from 31.7 to 33.1 μM in parental lines and from 30.2 to 38.5 μM in resistant counterparts (Fig. 6a). Western blot analysis demonstrated increased expression of ABCC1 in resistant line HCC1954-TDXdR compared to the respective parental line (Fig. 6b and Supplementary Fig. S10), supporting a potential role of ABCC1 in mediating resistance to T-DXd; the expression was comparable in the SUM190 resistant and parent lines.

Further, combination treatments of MK-571 with T-DXd significantly reduced cell viability in HCC1954-TDXdR cells: co-treatment with MK-571 (20 μM) and T-DXd (133 nM) led to a significant reduction in viability compared to either agent alone (*p* < 0.0001, Fig. 6c). In SUM190-TDXdR cells, the combination produced a slight viability decrease, with MK-571 alone having little effect. The results in HCC1954 parent/resistant cell lines support the hypothesis that ABCC1-mediated drug efflux contributes to resistance to T-DXd, and its inhibition by MK-571 restores T-DXd sensitivity; the results seen in SUM190 lines suggest potential variations in resistance mechanisms within different contexts and warrant further investigation.

## Discussion

T-DXd has significantly improved the outcomes of patients with metastatic breast cancer, both in patients traditionally considered HER2-positive as well as in HER2-low patients^19–21^. Nevertheless, a significant percentage of patients exhibit a priori resistance, and virtually all ultimately develop resistance to this agent. While multiple mechanisms of resistance have been suggested based on preclinical studies and limited clinical studies, there have been few large clinical studies exploring T-DXd resistance^4,22^.

To address this need, we explored clinical outcomes in a large real-world database of patients treated with T-DXd, whose tumors were profiled using NGS and HER2 IHC/CISH. To the best of our knowledge, this is the largest breast cancer dataset of T-DXd-treated patients to undergo such evaluations. Our basic approach was to follow the passage of T-DXd through breast cancer cells as illustrated in Fig. 1, evaluating the expression of genes associated with cell surface targeting, internalization, intracellular trafficking, lysosomal degradation, topoisomerase inhibition, and drug efflux from the cell. We also evaluated the expression of genes associated with the immune microenvironment. Our findings imply three important mediators of T-DXd resistance: (1) HER2 (*ERBB2*) target levels or mutations, potentially affecting both T-DXd binding and ADCC; (2) ABCC1-mediated efflux and mutations in genes regulating ABCC1 levels (*NEF2L2*, *KEAP1*); and (3) mutations in the gene for the target of the toxic payload of T-DXd, topoisomerase (*TOP1*) (Fig. 1).

Our work suggests that expression of only a limited number of genes predicts OS in T-DXd-treated patients. Pre-eminent among these is HER2 (*ERBB2*), whether measured at the protein (IHC) or transcript (NGS) level. At the protein level, median OS progressively improved with increased extracellular target expression, increasing from 10.8 months (HER2-null) to 12.7 months (HER2 ultra-low) to 17.5 months (HER2-low) to 27.3 months (HER2-positive); these results are highly consistent with the results of the phase 2 DAISY trial^4^. Similarly, we found that *ERBB2* gene expression also predicts OS, with the greatest benefit seen in the highest quartile of expression.

When comparing transcript level with protein expression, classic IHC HER2-positive tumors predominantly fall within the top quartile of RNA expression. However, so-called HER2-low tumors show a wide range of *ERBB2* expression, comprising more than 20% of each of the four quartiles. Similarly, there is considerable variability in *ERBB2* transcript levels among HER2-null, HER2-ultra-low, and HER2-low tumors, suggesting a degree of fluidity and uncertainty of these designations.

In addition to HER2/*ERBB2*, *ABCC1* gene expression appeared to have a moderate but significant effect on outcome in T-DXd-treated patients. *ABCC1* (also known as MRP1) is a drug efflux pump that has been suggested to be upregulated in cells that are resistant to ADCs, leading to lower intracellular drug levels for shorter periods of time^23^. Considering the combination of HER2 and *ABCC1*, drug resistance might be considered as—in classic pharmacokinetic terms—a function of intracellular concentration (related to HER2) times time (related to ABCC1 drug efflux of T-DXd). Consistent with this concept, we show that the combination of high HER2 and low *ABCC1* is associated with the best outcomes, and *ABCC1* status stratifies patient outcome within each HER2 category. These findings are also consistent with prior preclinical studies showing that low HER2 levels and high *ABCC1* expression mediate resistance to another HER2-targeted ADC, T-DM1^24–26^, and may also suggest ABCC1 as a potential therapeutic target. While previous studies have implicated ABC transporters in resistance to various ADCs^23,27–29^, evidence linking a specific pump to T-DXd resistance has remained elusive. Our comprehensive survey of ABC transporter family gene expression identified *ABCC1* as an efflux pump that may have particular relevance in mediating T-DXd resistance in breast cancer, offering a strong rationale for its therapeutic targeting. We acknowledge the role other efflux pumps may also play. A recent study in a cohort of 179 patients with breast cancer identified higher expression of both *ABCB1* and *ABCC1* as associated with shorter duration of T-DXd treatment, and *ABCB1* was also associated with shorter T-DXd-specific OS^30^. However, our extensive survey of all known efflux pumps in the large cohort of T-DXd treated breast tumors showed that *ABCB1* expression was not significantly associated with T-DXd OS in either multivariate or univariate analysis, in sharp contrast to *ABCC1* expression.

Examining the mutational pressures induced by T-DXd treatment, we conclude that *ERBB2* somatic mutations represent a rational potential mechanism of resistance to T-DXd. Previous studies have demonstrated that breast cancers with these mutations are sensitive to (some) kinase inhibitors but not to monoclonal antibody-based therapies, and indeed often lack an external HER2 membrane domain^31,32^. Similarly, *NFE2L2* and *KEAP1* mutations represent rational resistance mechanisms for T-DXd through their impact on *ABCC1* expression^16,17^. Interestingly, preclinical studies have shown that camptothecin, a tecan-like topoisomerase I inhibitor, suppresses *NFE2L2* expression^33,34^. Therefore, prolonged exposure to T-DXd may select for mutations in the NFE2L2/KEAP1 pathway—as we observed in our study (Fig. 6B, C)—thereby circumventing this particular mechanism of increased drug sensitivity.

Although these results present plausible resistance mechanisms supported by our clinical outcome findings, there are limitations to this post-T-DXd enrichment analysis. First, patients were exposed to a variety of other therapies at various timepoints that may confound the effects of T-DXd (Supplementary Table S5). Second, we did not have matched patient samples to examine mutational pressures at the individual patient level and therefore cannot establish causality between T-DXd treatment and the emergence of the identified mutations. However, in our large cohort of unpaired samples, we observed expected and previously reported events associated with T-DXd resistance—such as *TOP1* mutations^14^—supporting the validity of our findings. Third, we did not observe differences in *ABCC1* levels based on the presence or absence of *NFE2L2* or *KEAP1* mutations (although this analysis was not well powered). Expression of *ABCC1* can be regulated by methylation status that might be relevant in treatment-resistant breast cancers, potentially enabling expression changes without upstream genetic alterations^35^. In addition, post-transcriptional regulation by microRNAs (e.g., miR-326, miR-133a) may modulate *ABCC1* abundance and function, influencing drug efflux independently of genomic events^36,37^. Therefore, *ABCC1* upregulation and T-DXd resistance may occur via multiple converging mechanisms, of which NRF2–KEAP1 dysregulation represents only one pathway. Systematic functional studies will be needed to dissect these diverse regulatory routes and their relative clinical importance. An additional limitation to our study is the limited in vitro mechanistic evidence. While results in one cell line support our conclusions regarding ABCC1, the lack of effect of ABCC1 inhibition on viability of SUM190-TDXdR cells points to context-dependent resistance mechanisms that may not be overcome by blocking ABCC1. Furthermore, we only used HER2+ cell line models and thus cannot infer a similar effect in the context of HER2-low or HER2-ultra-low. Finally, a limitation of real-world data is that we are restricted to information available in insurance claims databases. As claims data lack standard trial endpoints and treatment discontinuation details, treatment-associated OS was used to assess T-DXd benefit as a more objective and clinically meaningful endpoint.

In conclusion, our work suggests that the combination of reduced HER2 cell surface expression and increased *ABCC1*-mediated efflux is associated with resistance to T-DXd in metastatic breast cancer. It will be of interest to see whether similar resistance patterns occur in other HER2-targeting scenarios, given the recent tissue-agnostic approval of T-DXd for HER2-positive solid tumors by the US Food and Drug Administration^38^. Future work could involve expanded cohorts and unbiased transcriptomic profiling to place our findings on *ABCC1* within the context of broader resistance pathways and identify additional transcriptional programs associated with T-DXd outcomes. The identification of resistance mechanisms might lead to improved precision of administration of this agent and could lead to novel therapeutic approaches to overcome resistance.

## Methods

### Patient samples

A total of 2799 T-DXd-treated breast cancer samples that underwent comprehensive tumor profiling at Caris Life Sciences (Phoenix, AZ, USA) were included in our analysis. No matched samples were available for analysis. This study was conducted in accordance with the guidelines of the Declaration of Helsinki, the Belmont Report, and the US Common Rule. In accordance with 45 CFR 46.104(d)(4), this study was conducted using retrospective, de-identified clinical data and is considered exempt from Institutional Review Board (IRB) review, with a waiver of patient-informed consent. Waiver of patient consent and exempt status were determined by WCG IRB.

### Tumor profiling

Tumor profiling was performed in a CAP/CLIA-certified laboratory, and all results meet standards for clinical reporting. Formalin-fixed paraffin-embedded (FFPE) specimens underwent pathology review to measure percent tumor content and tumor size. Microdissection was performed on all tumor samples to enrich tumor content and ensure comparability across samples. RNA sequencing data were analyzed in the pre-T-DXd samples (*n* = 2420), and DNA sequencing data were analyzed in all samples (*n* = 2799).

For whole transcriptome sequencing (WTS), a minimum of 10% of tumor content in the area for microdissection was required. Qiagen RNA FFPE tissue extraction kit was used for extraction, and the RNA quality and quantity were determined using the Agilent TapeStation. Biotinylated RNA baits were hybridized to the synthesized and purified cDNA targets, and the bait-target complexes were amplified in a post-capture PCR reaction. WTS was performed on an Illumina NovaSeq 6000 system (Illumina, San Diego, CA, USA) (RRID:SCR_016387) to an average of 60 M reads. Raw data were demultiplexed by Illumina Dragen BioIT accelerator, trimmed, counted, and sequences aligned to human reference genome hg19 by Spliced Transcripts Alignment to a Reference (STAR) (RRID:SCR_004463)^39^. Transcripts per million (TPM) values were generated using the Salmon expression pipeline (RRID:SCR_017036)^40^. Interferon-gamma scores were calculated using an 18-gene signature previously shown to be associated with immune checkpoint inhibitor outcome in various cancer types^41^. The RNA deconvolution program QuanTISeq (RRID:SCR_022993)^42^ was used to infer cell populations in the tumor immune microenvironment.

Genomic DNA was sequenced using the NextSeq 500 (RRID:SCR_014983) or NovaSeq 6000 platforms (Illumina, Inc.). For NextSeq sequenced tumors, a custom-designed SureSelect XT assay was used to enrich 592 whole-gene targets (Agilent Technologies, Santa Clara, CA). For NovaSeq sequenced tumors, a panel covering more than 700 clinically relevant genes at high coverage and high read-depth was used, along with another panel designed to enrich for an additional >20,000 genes at lower depth. Variants detected were mapped to reference genome hg19 (592-gene panel) or hg38 (WES) using the Burrows-Wheeler Aligner (BWA 0.7.17) (RRID:SCR_010910) embedded in the analysis tools licensed from Sentieon®. Bioinformatic tools, including SamTools (RRID:SCR_002105), Pindel (RRID:SCR_000560), and snpEff (RRID:SCR_005191) were incorporated to perform variant calling functions, and annotations were standardized to the Human Genome Variation Society format. Filtering was performed to remove benign variants, low-quality and low-depth variants, or variants determined to be unreliable in several analyses, like strand bias and repeat analysis. The 5% variant allele frequency and five alignments supporting a variant were required to call positive variants. Board-certified molecular geneticists interpreted genetic variants identified according to the American College of Medical Genetics and Genomics (ACMG) standards.

HER2 immunohistochemistry (IHC) was performed using the VENTANA® Pathway anti-HER2/neu RxDx (4B5) antibody (Roche (Ventana), Tucson, AZ, USA). HER2 chromogenic in-situ hybridization (CISH) was performed using the INFORM HER-2 Dual ISH DNA Probe Cocktail (Roche (Ventana). IHC was performed on full FFPE sections of glass slides, and results were read by a board-certified pathologist; all scoring followed American Society of Clinical Oncology (ASCO)/College of American Pathologists (CAP) guidelines^10^ (Supplementary Table S1). HER2 status was determined by a combination of IHC and CISH: HER2-positive: IHC 3+ with >10% of cells positive; HER2-low: IHC 1+ with >10% of cells positive, or IHC 2+ with >10% of cells positive combined with a negative CISH result; HER2-ultra-low: IHC 1+, 2+, or 3+ with ≤10% of cells positive; HER2-null: IHC 0+.

### Statistical analysis

Real-world clinical data were obtained from insurance claims, which encompass detailed records of health services that patients received. This includes prescribed medications, performed procedures, and established diagnoses. Overall survival (OS) was calculated from tissue collection to the date of the patient’s last known clinical activity, while treatment-specific OS was defined as the period from the start of treatment with T-Dxd to last contact. Patient death information, when available, was used to calculate patient survival. For patients with no death dates on record, and there were no insurance claims for a period exceeding 100 days, it was inferred that the patient had deceased. Conversely, patients who had a documented clinical activity within 100 days prior to the latest data update were considered censored in the analysis. Kaplan–Meier survival estimates were generated for cohorts defined by molecular characteristics. The Hazard Ratio (HR) was computed utilizing the Cox proportional hazards model, and the significance of differences (*p* values) in survival times was assessed with the log-rank test. Multivariate analysis was conducted using RNA TPM inputs as continuous variables in a Cox-Proportional Hazard model, with significance determined at *p* < 0.05. Genes and RNA signatures surveyed for correlation with patient outcomes are shown in Supplementary Table 2. Molecular comparisons were performed using Chi-square tests, and *p* values were adjusted for multiple comparisons using the Benjamini-Hochberg method (*q* < 0.05).

### Preclinical studies in cell lines

The HER2-positive breast cancer cell lines HCC1954 and SUM190 were obtained from the American Type Culture Collection (ATCC, Manassas, VA, USA) and BioIVT (Westbury, NY, USA), respectively. HCC1954 cells were cultured in RPMI-1640 medium (GenDEPOT, Katy, TX, USA) supplemented with 10% fetal bovine serum (FBS; GenDEPOT) and 1% antibiotic-antimycotic solution (GenDEPOT). SUM190 cells were maintained in Ham’s F-12 medium (Thermo Fisher, Waltham, MA, USA) supplemented with 5% FBS, 1% antibiotic-antimycotic, 5 µg/mL insulin (Sigma-Aldrich, St. Louis, MO, USA), and 1 µg/mL hydrocortisone (Sigma-Aldrich). All cell lines were authenticated by short tandem repeat (STR) profiling using primer extension to detect single-base deviations and were confirmed to be free of mycoplasma contamination using the MycoAlert™ Mycoplasma Detection Kit (Lonza, Morristown, NJ, USA).

T-DXd-resistant cell lines (HCC1954-TDXdR and SUM190-TDXdR) were utilized alongside their parental counterparts to evaluate ABCC1 expression and assess the effect of ABCC1 inhibition on reversing T-DXd resistance. As previously characterized, the T-DXd resistant cell lines display reduced *ERBB2* gene copy number amplification and reduced HER2 protein and cell surface expression compared to their sensitive counterparts, while retaining dose-dependent growth inhibition to free DXd^18^. Sulforhodamine B (SRB) was obtained from Sigma–Aldrich, and the ABCC1 inhibitor MK-571 was purchased from MedChemExpress (Monmouth Junction, NJ, USA). For cell viability assays, cells were seeded in 96-well plates and treated with serial dilutions of MK-571, T-DXd, or their combination for 10 days. Cell viability was quantified using the SRB assay, and absorbance was measured using a Tecan Spark microplate reader (Baldwin Park, CA, USA). Viability was expressed as a percentage relative to untreated control cells. Half-maximal inhibitory concentration (IC_50_) values were calculated using non-linear regression analysis in GraphPad Prism (version 10.0). ABCC1 protein levels were evaluated by Western blotting. Total protein lysates were prepared from both parental and resistant cell lines using RIPA buffer supplemented with protease inhibitors (GenDEPOT). Protein concentration was determined using the BCA assay (Thermo Fisher). Equal amounts of total protein (25 µg) were separated by SDS-PAGE and transferred onto polyvinylidene difluoride (PVDF) membranes. Membranes were probed with primary antibodies against ABCC1 (Clone:1G4A2, Proteintech, Rosemont, IL, USA) and β-actin as loading control (Clone: AC-15, Sigma–Aldrich), followed by appropriate HRP-conjugated secondary antibodies (Thermo Fisher). Protein bands were detected using enhanced chemiluminescence and visualized with the Azure 500 imaging system (Azure Biosystems, Dublin, CA, USA).

## Supplementary information

Supplementary Information

## Data Availability

The deidentified sequencing data are owned by Caris Life Sciences and cannot be publicly shared due to the data usage agreement in place. These data will be made available to researchers for replication and verification purposes through our letter of intent process, which is generally fulfilled within 6 months. For more information on how to access this data, please contact Joanne Xiu at [jxiu@carisls.com](mailto:jxiu@carisls.com).
