# Supplementary material for: Mechanisms of resistance to trastuzumab deruxtecan in breast cancer elucidated by multi-omic molecular profiling
Source: NPJ Breast Cancer. 2025 Dec 20;12:1. doi: 10.1038/s41523-025-00868-y (PMC12774910; doi:10.1038/s41523-025-00868-y)

Mechanisms of Resistance to Trastuzumab Deruxtecan in Breast Cancer Elucidated by Multi-omic Molecular Profiling

[1]George W. Sledge, [1]Joanne Xiu, [2]Reshma L. Mahtani, [2]Ana C. Sandoval Leon, [3] Funda Meric-Bernstam, [1]Jennifer R. Ribeiro, [1]Ninad Kulkarni, [4]Dileep R. Rampa, [4]Jangsoon Lee, [4]Naoto T. Ueno, [1]Matthew J. Oberley, [1]Milan Radovich, [1]David B. Spetzler

[1]Caris Life Sciences, Phoenix, AZ, USA
[2]Miami Cancer Institute, Miami, FL, USA
[3]MD Anderson Cancer Institute, TX, USA
[4]University of Hawai'i Cancer Center, HI, USA

Corresponding Author:
George W. Sledge, Jr.
Caris Life Sciences
4610 S 44th Pl,
Phoenix, AZ 85040
(469) 724-6502
gsledge@carisls.com
ORCID: 0000-0003-0297-0775

Supplementary Materials

Supplementary Table S1: HER2 categorization based on IHC/CISH results

IHC Intensity	IHC Percentage	CISH result	HER2 IHC ASCO Category	HER2 Category
0	0	N/A	0, negative	HER2-null
1/2/3+	=<10%	N/A	0, negative	HER2-ultra-low
1+	>10%	N/A	1+, negative	HER2-low
2+	>10%	CISH Neg	2+, equivocal	HER2-low
2+	>10%	CISH Pos	2+, equivocal	HER2-positive
3+	>10%	N/A	3+, Positive	HER2-positive

Supplementary Table S2: RNA signatures and genes surveyed in multivariate analysis

RNA Signatures	Pathway	Gene	Pathways	Gene	Pathways
Interferon Gamma Signature	TME	*ABCA1*	ABC Transporter	*GZMB*	ADCC pathway
QuanTISeq-B cell	TME	*ABCA10*	ABC Transporter	*FCGR1A*	ADCC pathway
QuanTISeq-Macrophage M1	TME	*ABCA11P*	ABC Transporter	*FCGR1B*	ADCC pathway
QuanTISeq-Macrophage M2	TME	*ABCA12*	ABC Transporter	*FCGR1C*	ADCC pathway
QuanTISeq-Neutrophil	TME	*ABCA2*	ABC Transporter	*FCGR2A*	ADCC pathway
QuanTISeq-NK cell	TME	*ABCA3*	ABC Transporter	*FCGR2B*	ADCC pathway
QuanTISeq-T cell CD4+	TME	*ABCA4*	ABC Transporter	*FCGR2C*	ADCC pathway
QuanTISeq-T cell CD8+	TME	*ABCA5*	ABC Transporter	*FCGR3A*	ADCC pathway
QuanTISeq-T cell regulatory	TME	*ABCA6*	ABC Transporter	*FCGR3B*	ADCC pathway
QuanTISeq-Myeloid dendritic cell	TME	*ABCA7*	ABC Transporter	*PAK4*	Cytoskeleton organization
		ABCA8	ABC Transporter	*CAV1*	Endocytosis
		ABCA9	ABC Transporter	*CAV2*	Endocytosis
		ABCB10	ABC Transporter	*RAB5B*	Endocytosis
		ABCB4	ABC Transporter	*RAB6A*	Intracellular trafficking
		ABCB6	ABC Transporter	*RAB6B*	Intracellular trafficking
		ABCB7	ABC Transporter	*ATG9A*	Lysosome pathway
		ABCB8	ABC Transporter	*HTT*	Lysosome pathway
		ABCB9	ABC Transporter	*CTSA*	Lysosome pathway
		ABCC10	ABC Transporter	*CTSB*	Lysosome pathway
		ABCC11	ABC Transporter	*CTSC*	Lysosome pathway
		ABCC3	ABC Transporter	*CTSD*	Lysosome pathway
		ABCC8	ABC Transporter	*CTSF*	Lysosome pathway
		ABCC9	ABC Transporter	*CTSH*	Lysosome pathway
		ABCD1	ABC Transporter	*CTSK*	Lysosome pathway
		ABCD3	ABC Transporter	*CTSL1*	Lysosome pathway
		ABCD4	ABC Transporter	*CTSO*	Lysosome pathway

		ABCE1	ABC Transporter	*CTSS*	Lysosome pathway
		ABCF1	ABC Transporter	*CTSL2*	Lysosome pathway
		ABCF2	ABC Transporter	*CTSW*	Lysosome pathway
		ABCF3	ABC Transporter	*CTSZ*	Lysosome pathway
		ABCG1	ABC Transporter	*LGMN*	Lysosome pathway
		ABCB1	ABC Transporter	*ESR1*	Prognostic Marker
		ABCC1	ABC Transporter	*PGR*	Prognostic Marker
		ABCG2	ABC Transporter	*MKI67*	Prognostic Marker
		ABCC2	ABC Transporter	*EGFR*	Target Dimerization Partner
		ABCC4	ABC Transporter	*NRG1*	Target Dimerization Partner
		ABCC5	ABC Transporter	*ERBB2*	Target Dimerization Partner
		ABCC6	ABC Transporter	*ERBB3*	Target Dimerization Partner
				ERBB4	Target Dimerization Partner
				TOP1	Topoisomerase
				TOP2A	Topoisomerase
				TOP3B	Topoisomerase
				TOP2B	Topoisomerase
				TUBB	Tubulin
				TUBB2A	Tubulin
				TUBB3	Tubulin
				TUBB4B	Tubulin
				TUBB6	Tubulin

Supplementary Table S3: Cox Proportional Regression analysis of genes and gene interactions highly correlated with *ABCC1*.

Genes and Interactions	Correlation with *ABCC1*		Cox Proportional Regression
	**Spearman ρ**	***p*-value**	**"-log10(p value)"**
ABCC1	1	N/A	2.14
ABCE1	0.601	< 0.0001	1.713
ABCB7	0.6149	< 0.0001	0.629
ABCF2	0.6388	< 0.0001	0.12
*ABCE1*ABCC1*	N/A	N/A	0.104
*ABCB7*ABCC1*	N/A	N/A	0.095
*ABCF2*ABCC1*	N/A	N/A	0.056

Supplementary Table S4: Testing *ABCC1* thresholds for association with T-DXd-associated overall survival. Bolded row represents the threshold of selection for follow-up analyses.

***ABCC1* Threshold (%)**	**HR**	**HR Lower 95% CI**	**HR Upper 95% CI**	**Log-rank p-value**	**N High**	**N Low**	**Median Survival High (months)**	**Median Survival Low (months)**
5 percentile	0.8095	0.5872	1.1161	0.1969	1737	92	16.5	28.0
10 percentile	0.8271	0.6556	1.0436	0.1095	1646	183	16.5	23.5
15 percentile	0.7948	0.6538	0.9663	0.0211	1554	275	16.2	23.4
20 percentile	0.7815	0.6556	0.9316	0.0059	1463	366	15.7	23.1
25 percentile	0.8353	0.7133	0.9781	0.0254	1371	458	15.7	22.0
30 percentile	0.8401	0.7244	0.9743	0.0212	1280	549	15.7	21.5
35 percentile	0.838	0.7272	0.9656	0.0146	1189	640	15.6	21.5
40 percentile	0.8068	0.7032	0.9258	0.0022	1097	732	15.4	20.6
45 percentile	0.8287	0.7249	0.9474	0.0059	1006	823	15.5	19.5
50 percentile	0.8198	0.7183	0.9355	0.0032	914	915	15.4	19.3
55 percentile	0.8482	0.7435	0.9676	0.0142	823	1006	15.4	18.2
60 percentile	0.8706	0.7623	0.9943	0.0410	732	1097	15.5	17.9
65 percentile	0.8415	0.7351	0.9634	0.0124	640	1189	15.3	17.9
70 percentile	0.8382	0.7293	0.9635	0.0130	549	1280	15.3	17.9
**75 percentile**	**0.7961**	**0.6883**	**0.9207**	**0.0021**	**457**	**1372**	**14.2**	**17.8**
80 percentile	0.7918	0.6778	0.9249	0.0032	366	1463	13.3	17.6
85 percentile	0.7874	0.6638	0.934	0.0060	275	1554	13.4	17.3
90 percentile	0.7738	0.6352	0.9428	0.0108	183	1646	14.0	17.1
95 percentile	0.8091	0.6223	1.052	0.1132	92	1737	14.2	17.0

Supplementary Table S5: Clinical and demographic characteristics of cohort stratified by *ABCC1* quartiles

		ABCC1 Q1	*ABCC1* Q2	*ABCC1* Q3	*ABCC1* Q4	Total	Adjusted p-value (BH)
**Gender**	**Female**	456 (25%)	466 (26%)	445 (25%)	439 (24%)	1806 (100%)	>0.1
	**Male**	7 (30%)	4 (17%)	9 (39%)	3 (13%)	23 (100%)	>0.1
**Age**	**Median**	58	56	58	59	58	>0.1
	**IQR**	49-66	48-65	49-65	49-66	49-66	>0.1
**HER2**	**Her2 Positive**	92 (29%)	87 (27%)	71 (22%)	72 (22%)	322 (100%)	>0.1
	**Her2 Low**	145 (25%)	147 (25%)	150 (26%)	137 (24%)	579 (100%)	>0.1
	**Her2 Ultra Low**	81 (24%)	76 (23%)	85 (25%)	93 (28%)	335 (100%)	>0.1
	**Her2-null**	74 (22%)	82 (25%)	86 (26%)	90 (27%)	332 (100%)	>0.1
	**Her2 Other/Unknown**	71 (27%)	78 (30%)	62 (24%)	50 (19%)	261 (100%)	>0.1
**HR**	**HR Positive**	286 (25%)	286 (25%)	295 (26%)	273 (24%)	1140 (100%)	>0.1
	**HR Negative**	112 (24%)	113 (25%)	111 (24%)	125 (27%)	461 (100%)	>0.1
	**NA**	65 (29%)	71 (31%)	48 (21%)	44 (19%)	228 (100%)	>0.1
**RACE**	**Asian or Pacific Islander**	18 (22%)	19 (23%)	25 (30%)	21 (25%)	83 (100%)	>0.1
	**Black or African American**	65 (27%)	63 (26%)	62 (25%)	54 (22%)	244 (100%)	>0.1
	**White**	264 (25%)	264 (25%)	244 (24%)	265 (26%)	1037 (100%)	>0.1
	**Other**	16 (25%)	20 (31%)	11 (17%)	17 (27%)	64 (100%)	>0.1
	**Unknown**	100 (25%)	104 (26%)	112 (28%)	85 (21%)	401 (100%)	>0.1
**ETHNICITY**	**Hispanic or Latino**	42 (29%)	38 (26%)	33 (22%)	34 (23%)	147 (100%)	>0.1
	**Not Hispanic or Latino**	328 (25%)	321 (25%)	324 (25%)	329 (25%)	1302 (100%)	>0.1
	**Unknown**	93 (24%)	111 (29%)	97 (26%)	79 (21%)	380 (100%)	>0.1
**Specimen Site**	**Breast**	112 (22%)	137 (27%)	135 (26%)	131 (25%)	515 (100%)	>0.1
	**Liver**	102 (25%)	113 (27%)	95 (23%)	103 (25%)	413 (100%)	>0.1
	**Lymph Node**	41 (20%)	58 (28%)	59 (29%)	48 (23%)	206 (100%)	>0.1
	**Bone**	48 (31%)	31 (20%)	35 (22%)	42 (27%)	156 (100%)	>0.1
	**Chest/Chest Wall**	23 (22%)	25 (24%)	29 (28%)	28 (27%)	105 (100%)	>0.1
	**Lung**	26 (25%)	20 (19%)	31 (30%)	28 (27%)	105 (100%)	>0.1
	**Skin**	23 (29%)	24 (31%)	15 (19%)	16 (21%)	78 (100%)	>0.1
	**Brain/CNS**	30 (40%)	24 (32%)	14 (19%)	7 (9%)	75 (100%)	0.025
	**GYN**	9 (45%)	7 (35%)	3 (15%)	1 (5%)	20 (100%)	>0.1
	**Connective Tissue**	5 (17%)	6 (20%)	9 (30%)	10 (33%)	30 (100%)	>0.1
	**Other Visceral Mets**	30 (45%)	11 (17%)	15 (23%)	10 (15%)	66 (100%)	0.025
	**Unclear/Other Specimen Site**	7 (20%)	9 (26%)	7 (20%)	12 (34%)	35 (100%)	>0.1

	**Other Non-Visceral Mets**	7 (28%)	5 (20%)	7 (28%)	6 (24%)	25 (100%)	>0.1
**PreCollection Therapy**	**Hormone Therapy**	313 (28%)	291 (26%)	256 (23%)	268 (24%)	1128 (100%)	>0.1
	**Chemotherapy**	234 (25%)	227 (25%)	227 (25%)	236 (26%)	924 (100%)	>0.1
	**Radiation Therapy**	228 (28%)	201 (24%)	200 (24%)	194 (24%)	823 (100%)	>0.1
	**Monoclonal Antibody**	165 (26%)	156 (25%)	151 (24%)	160 (25%)	632 (100%)	>0.1
	**Small Molecule Inhibitors**	111 (25%)	116 (26%)	108 (24%)	115 (26%)	450 (100%)	>0.1
	**Antibody-Drug Conjugate**	37 (16%)	51 (22%)	64 (28%)	78 (34%)	230 (100%)	0.015
	**Immune Checkpoint Inhibitors**	14 (18%)	17 (22%)	20 (26%)	27 (35%)	78 (100%)	>0.1
	**With Precollection therapy**	389 (26%)	373 (25%)	354 (24%)	361 (24%)	1477 (100%)	>0.1
	**No Precollection Therapy Recorded**	74 (21%)	97 (28%)	100 (28%)	81 (23%)	352 (100%)	>0.1
	**Total**	463 (0%)	470 (0%)	454 (0%)	442 (0%)	1829 (0%)	>0.1

Supplementary Figure S1: **Association of gene expression with T-DXd-specific overall survival (OS).** Kaplan-Meier evaluation of *MKI67I* **(a)**, *FCGR3A* **(b)**, *ABCA6* **(c)** and *RAB6A* **(d)** expression quartiles and association with T-DXd-specific OS, calculated from start of treatment with T-DXd to last patient contact using insurance claims data. 95% confidence intervals shown in parentheses. *p* value was calculated using log-rank test.

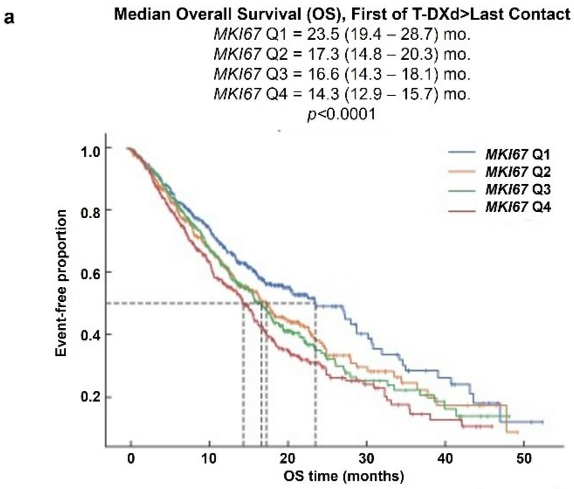

***MKI67* Q1**	454	261	75	25	12	2
***MKI67* Q2**	452	234	74	22	7	0
***MKI67* Q3**	455	234	70	19	7	0
***MKI67* Q4**	454	216	67	21	7	0

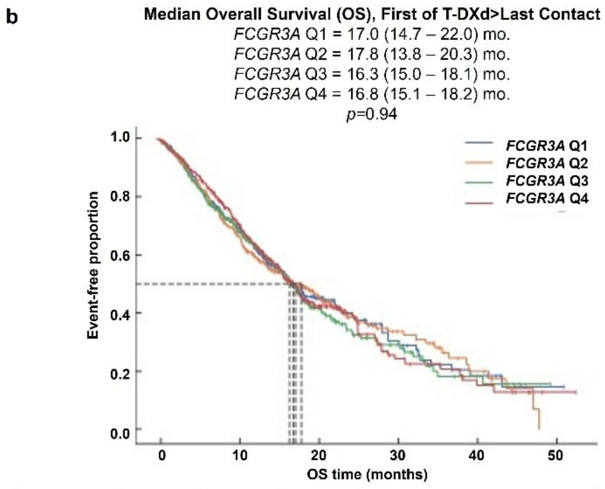

***FCGR3A* Q1**	456	242	69	21	9	1
***FCGR3A* Q2**	453	237	77	26	9	0
***FCGR3A* Q3**	455	223	69	23	7	0
***FCGR3A* Q4**	451	243	71	17	8	1

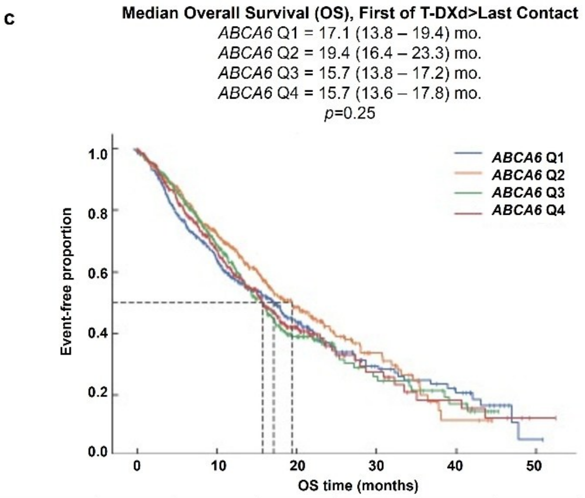

***ABCA6* Q1**	451	248	92	28	14	1
***ABCA6* Q2**	454	251	84	26	4	0
***ABCA6* Q3**	455	228	55	18	8	0
***ABCA6* Q4**	455	218	55	15	7	1

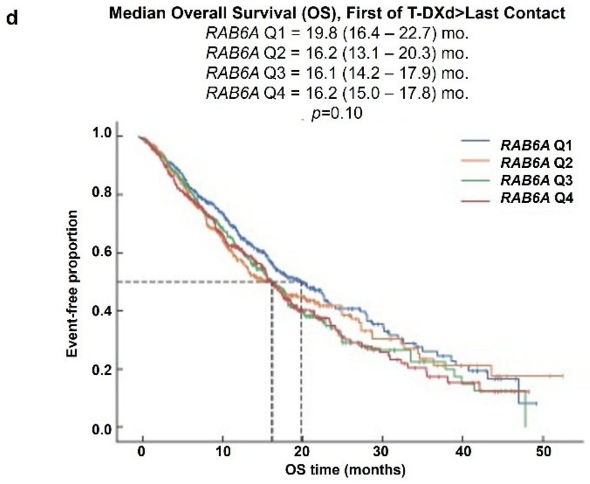

***RAB6A* Q1**	455	264	82	29	12	0
***RAB6A* Q2**	455	226	75	21	8	2
***RAB6A* Q3**	452	233	67	16	6	0
***RAB6A* Q4**	453	222	62	21	7	0

Supplementary Figure S2: Prognostic value of *KI67* gene expression in breast cancer. Kaplan−Meier curves show median overall survival (OS; tissue collection to last contact) for patients with *KI67*-high and *KI67*-low tumors (stratified by median TPM) treated with T-DXd **(a)** and not treated with T-DXd **(b).** HR, hazard ratio. 95% confidence intervals shown in parentheses. *p* value was calculated using log-rank test.

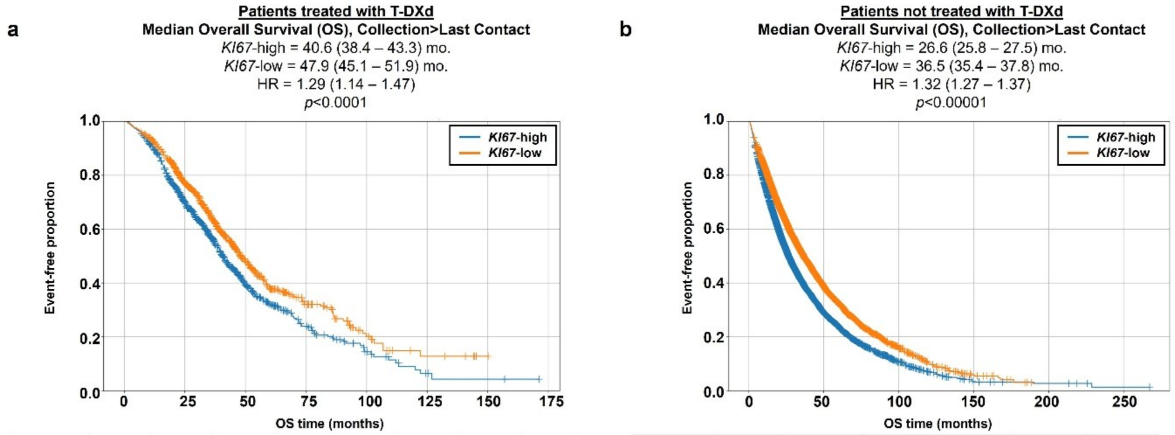

***KI67*-high**	954	565	154	48	17	4	2	0
***KI67*-low**	953	626	186	61	18	6	1	0

***KI67*-high**	9367	1180	111	10	5	1
***KI67*-low**	9366	1497	140	14	0	0

Supplementary Figure S3: Analysis of *ABCC1* expression in The Cancer Genome Atlas invasive breast cancer cohort (*N* = 1,084). (a) Percentage of tumors with mutations, structural variants, amplifications/deletions, or mRNA-high/low status for ABC genes. mRNA-high or mRNA-low status was determined by Z-scores relative to diploid expression. Oncoprint for *ABCC1* shown below. **(b)** Median mRNA expression for ABC genes in HER2 expression groups (equivocal, negative, positive). **(c)** Spearman rank correlation between *ABCC1* gene expression (RSEM, batch normalized from Illumina_HiSeq_RNASeqV2) and ABCC1 protein levels (mass spec by CPTAC). Data was obtained from cBioPortal (cbioportal.org).

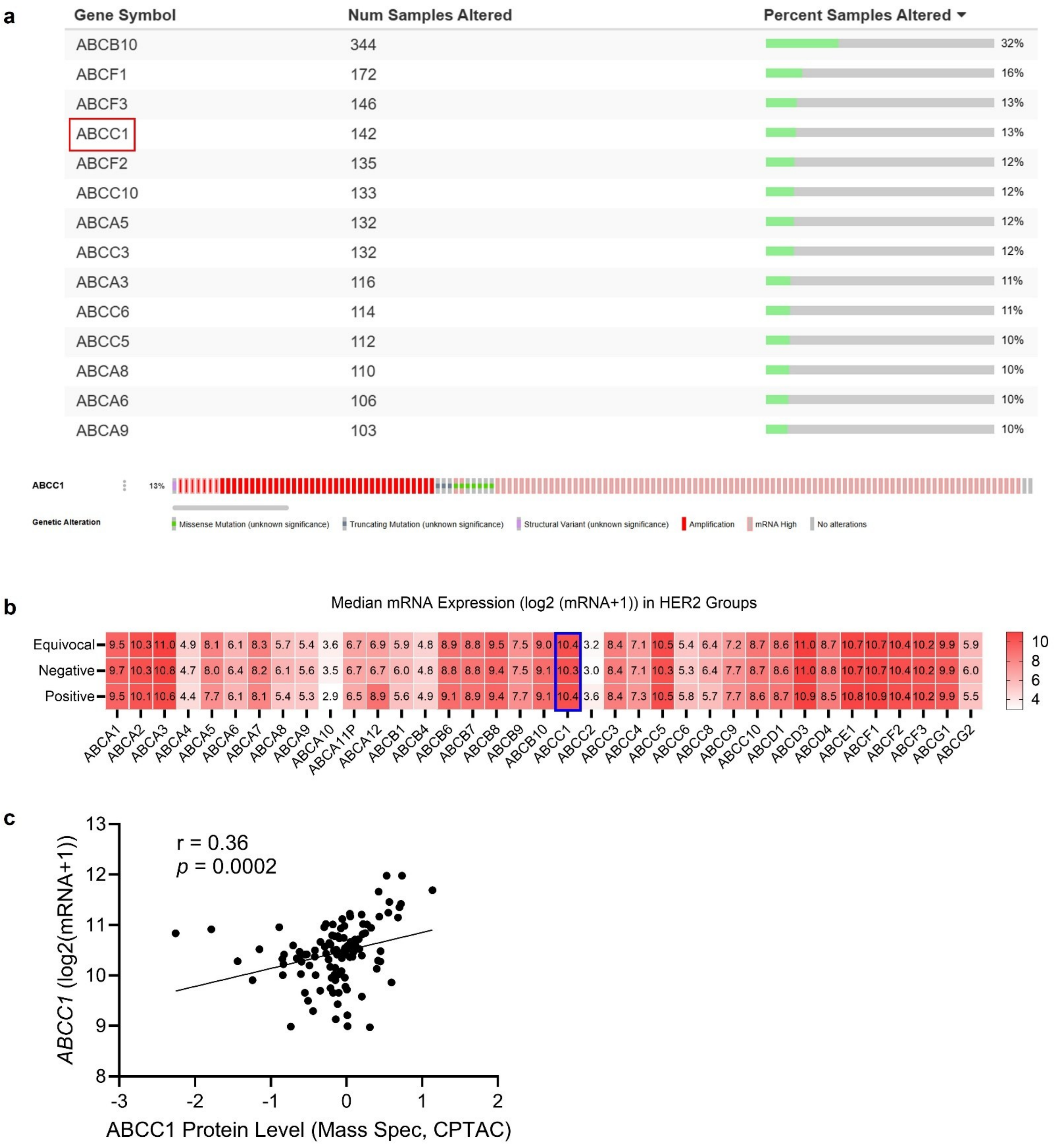

Supplementary Figure S4: Effect of *ABCC1* expression combined with HER2 categorization on T-DXd-specific overall survival (OS). OS (from start of T-DXd to last contact) was calculated from insurance claims. Kaplan-Meier analysis was performed to determine median OS for each molecularly defined group. 95% confidence intervals shown in parentheses. *p* value was calculated using log-rank test.

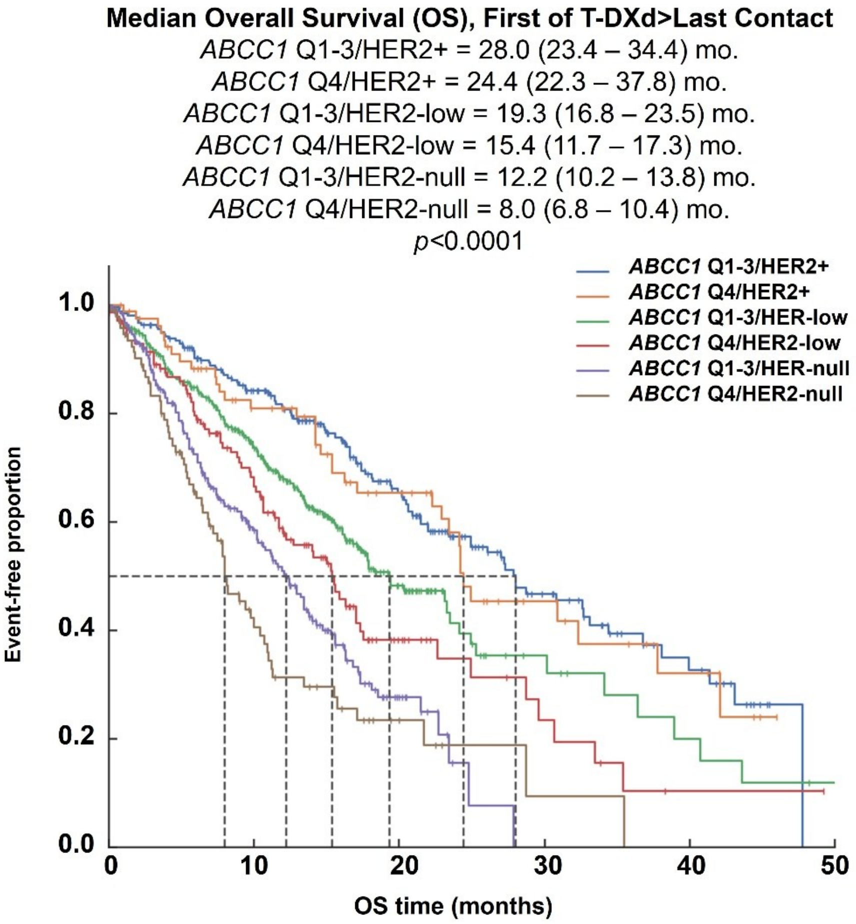

***ABCC1* Q1-3/HER2+**	243	175	95	38	14	0
***ABCC1* Q4/HER2+**	79	53	32	13	6	0
***ABCC1* Q1-3/HER-low**	435	226	54	11	5	2
***ABCC1* Q4/HER2-low**	137	73	17	6	1	0
***ABCC1* Q1-3/HER-null**	240	98	15	0	0	0
***ABCC1* Q4/HER2-null**	92	26	5	1	0	0

Supplementary Fig. S5: Prognostic value of *ABCC1* in breast cancer. Kaplan−Meier curves show median overall survival (OS; tissue collection to last contact) for *ABCC1*-high and *ABCC1*-low groups (stratified by median TPM) **(a)** and by expression quartiles **(b)**. HR, hazard ratio. 95% confidence intervals shown in parentheses.

a

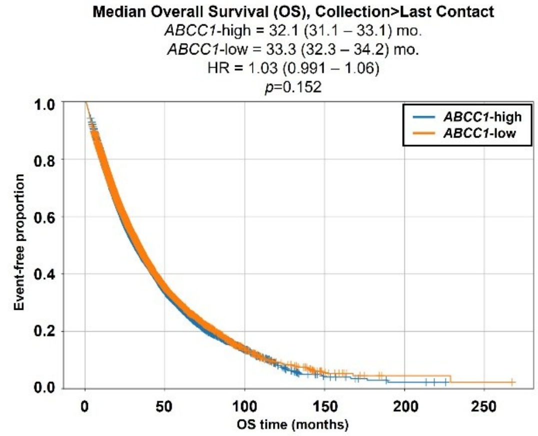

ABCC1-high	10320	1641	160	12	3	0
ABCC1-low	10320	1376	126	15	2	1

b

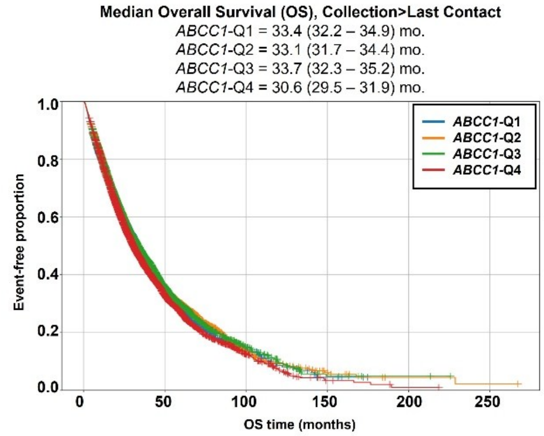

ABCC1-Q1	5160	619	54	4	0	0
ABCC1-Q2	5160	757	72	11	2	1
ABCC1-Q3	5160	788	78	6	2	0
ABCC1-Q4	5160	853	82	6	1	0

Supplementary Fig. S6: Expression of *ABCC1* (TPM) in pre- and post-treated specimens.
(a) Cohort treated with T-DXd. **(b)** Cohort treated with trastuzumab. ***, $p < 0.001$.

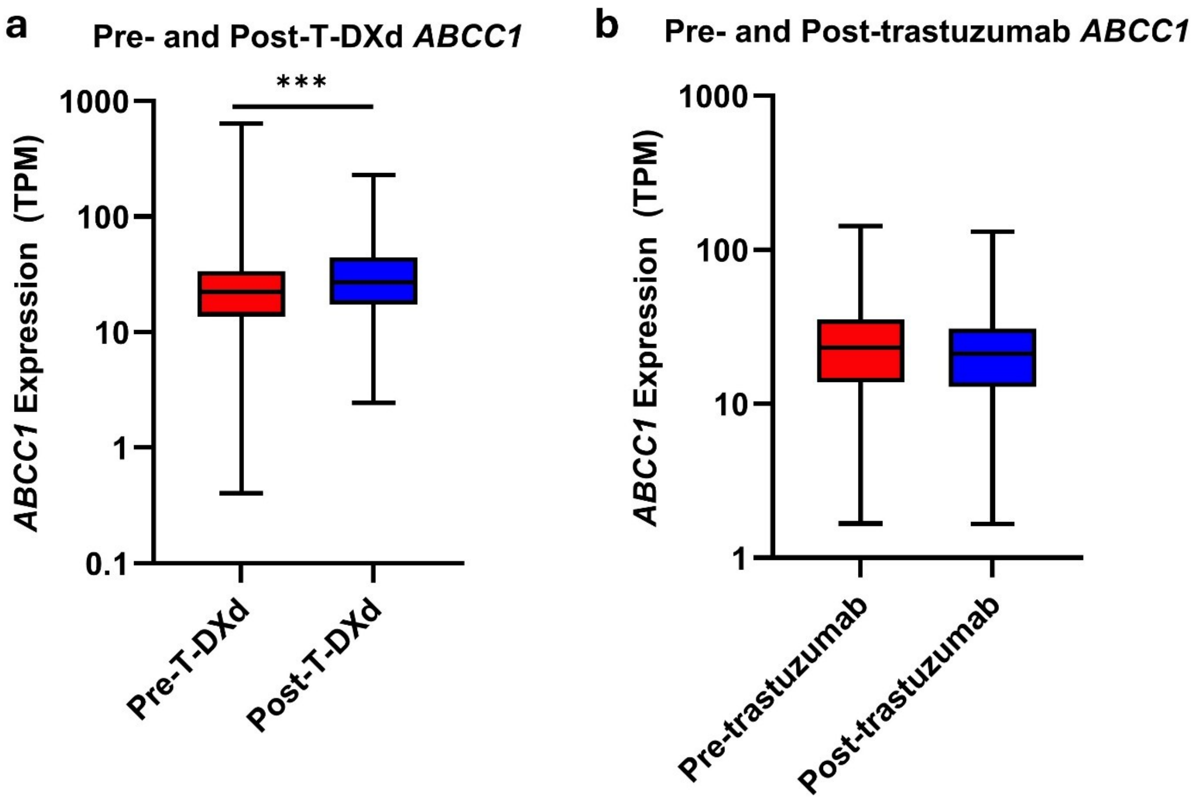

Supplementary Fig. S7: Oncoprint showing mutational landscape in pre- (right side of oncoprint) and post-T-DXd-treated (left side of oncoprint) specimens. Samples with mutations are indicated in red. Missing data are indicated in gray.

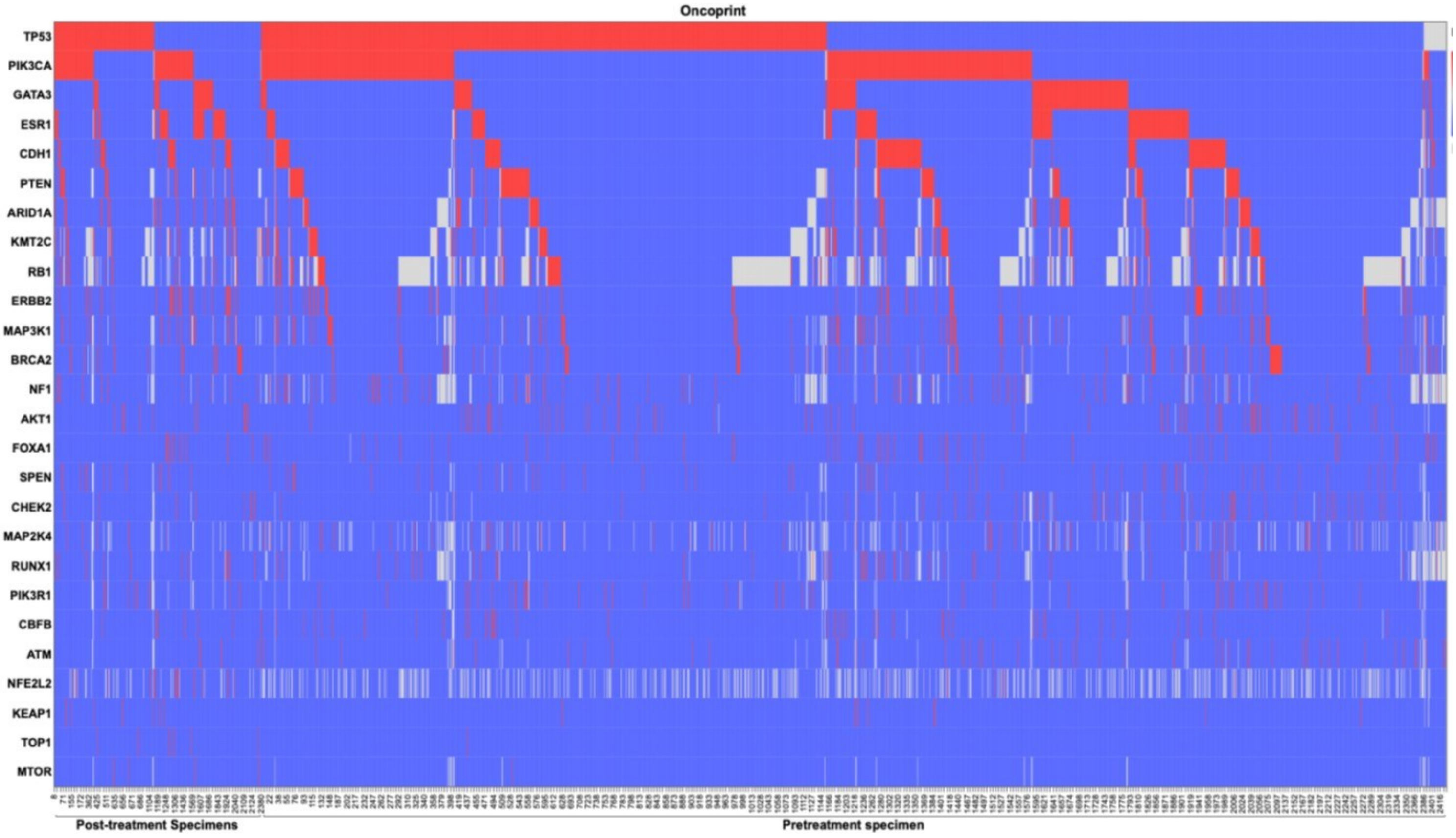

Supplementary Fig. S8: Lollipop plots of *ERBB2* and *TOP1* mutations in post-T-DXd specimens. (a) *ERRB2* pathogenic and likely-pathogenic mutations. *ERBB2* mutations are well annotated in the Caris database by board-certified molecular geneticists. Median *ERBB2* expression for kinase domain mutations was 119 TPM and was 76.9 TPM for ligand binding domain mutations (p = 0.02). **(b)** *TOP1* pathogenic mutations with protein changes shown below. *TOP1* mutations are rare events and not fully annotated in the Caris database. Interpretation of *TOP1* mutations shown was based on previous literature.

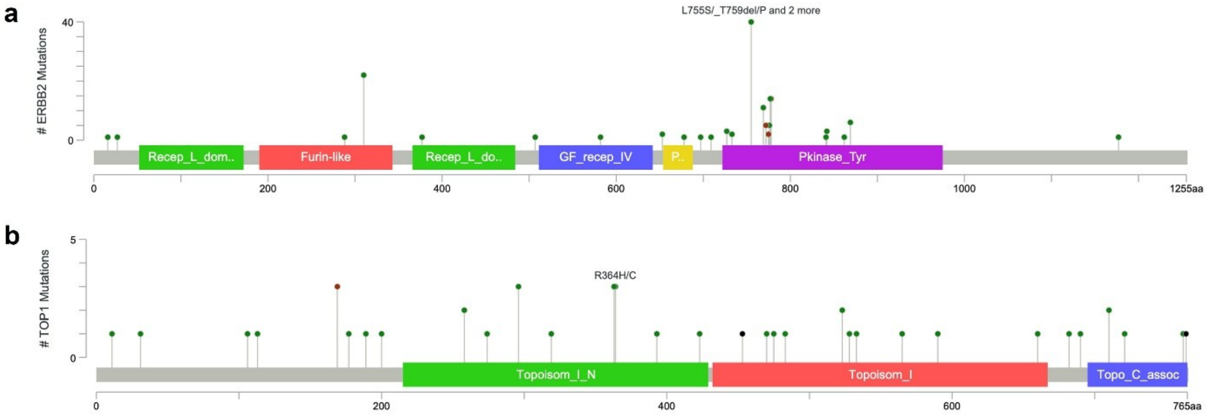

Biomarker	Protein Change	Interpreted Result	Technology	Interpretation
**TOP1**	D533N	Unclassified Variant	WES	Pathogenic
**TOP1**	E710K	Unclassified Variant	WES	Pathogenic
**TOP1**	E710K	Unclassified Variant	WES	Pathogenic
**TOP1**	G363C	Unclassified Variant	WES	Pathogenic
**TOP1**	G363C	Unclassified Variant	WES	Pathogenic
**TOP1**	G363C	Unclassified Variant	WES	Pathogenic
**TOP1**	R364H	Unclassified Variant	WES	Pathogenic
**TOP1**	R364H	Unclassified Variant	WES	Pathogenic

Supplementary Fig. S9: ***ABCC1*** **expression in** ***NFE2L2*** **and/or** ***KEAP1*** **mutant (MT) and wildtype (WT) tumors. (a)** All tumors. **(b)** Pre-T-DXd tumors. **(c)** Post-T-DXd tumors.

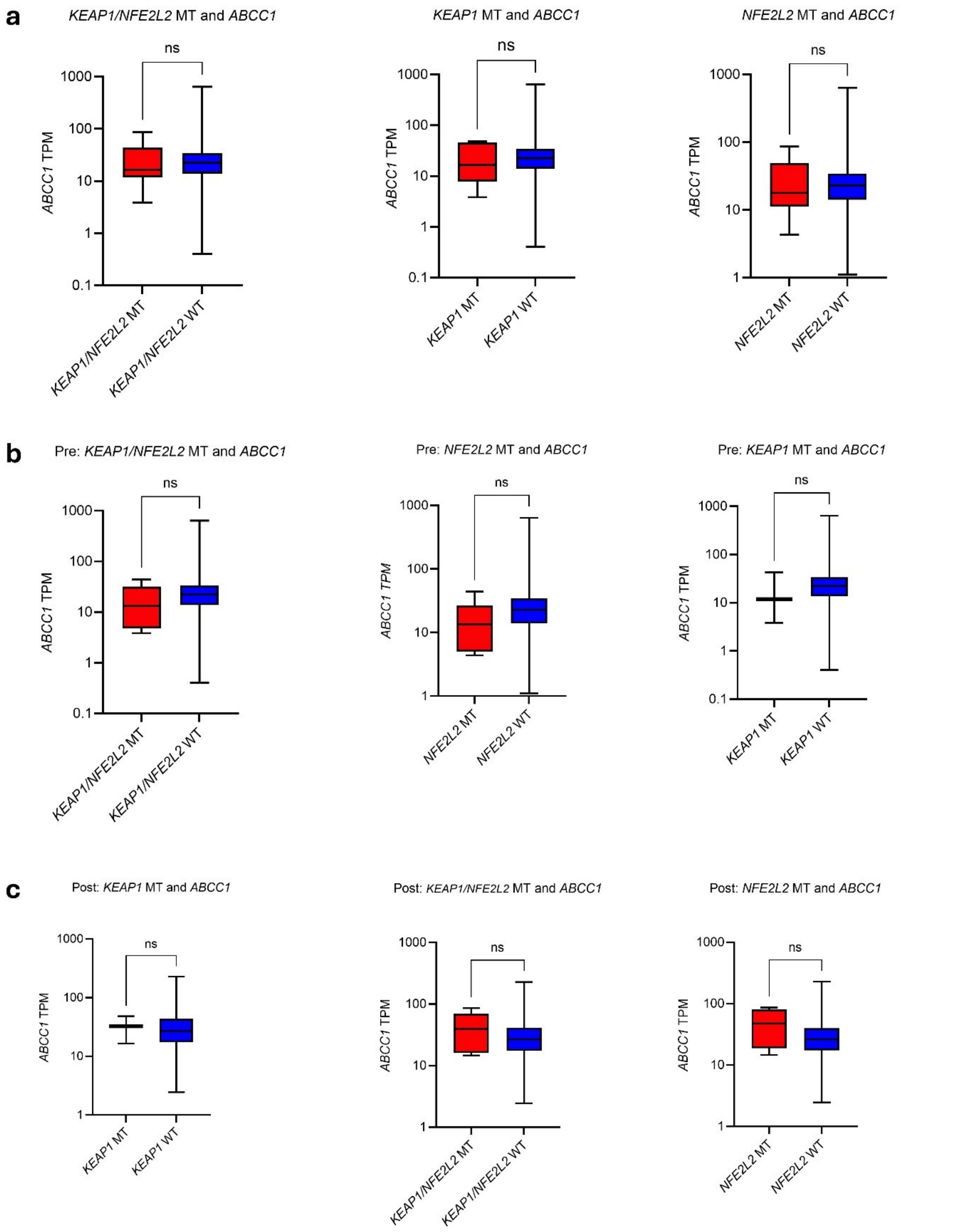

Supplementary Fig. S10: Uncropped blots for western blot images shown in Fig. 6b.
Uncropped blots include TNBC cell lines that are not included in this study.

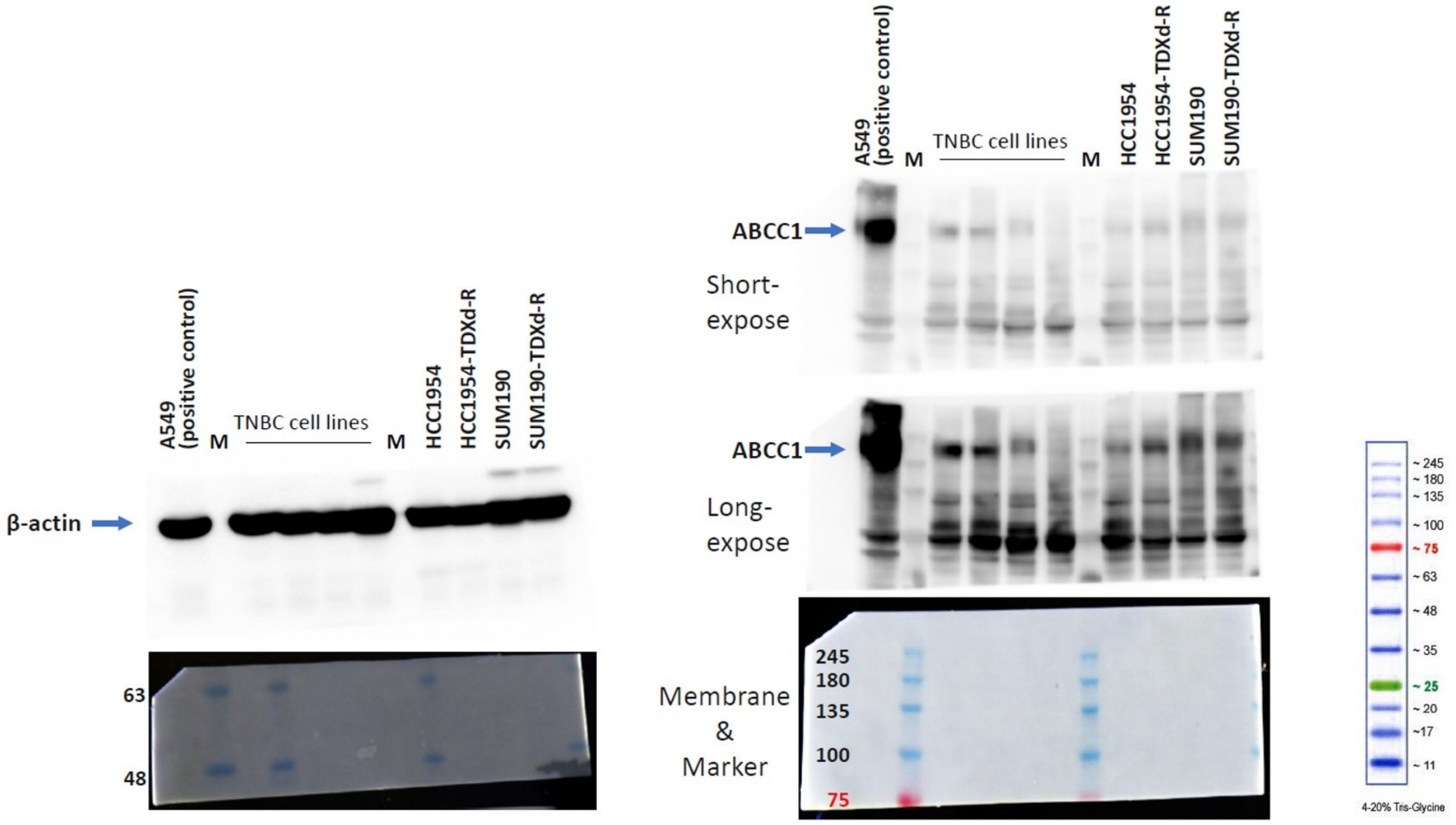

Supplement: Supplementary file 1 — Supplementary Information [file 41523_2025_868_MOESM1_ESM.pdf]
